# Cardiac Function After Cardiotoxic Treatments for Childhood Cancer—Left Ventricular Longitudinal Strain in Screening

**DOI:** 10.3389/fcvm.2021.715953

**Published:** 2021-10-18

**Authors:** Jussi Niemelä, Kaisa Ylänen, Anu Suominen, Kuberan Pushparajah, Sujeev Mathur, Taisto Sarkola, Kirsi Jahnukainen, Anneli Eerola, Tuija Poutanen, Kim Vettenranta, Tiina Ojala

**Affiliations:** ^1^Department of Pediatric Cardiology, Pediatric Research Center, New Children's Hospital, University of Helsinki, Helsinki University Hospital, Helsinki, Finland; ^2^Department of Pediatrics and Adolescent Medicine, Turku University Hospital, University of Turku, Turku, Finland; ^3^Center for Child Health Research, Tampere, Finland; ^4^Department of Pediatrics, Faculty of Medicine and Health Technology, Tampere University Hospital, Tampere University, Tampere, Finland; ^5^Division of Hematology-Oncology and Stem Cell Transplantation, New Children's Hospital, Pediatric Research Center, University of Helsinki, Helsinki University Hospital, Helsinki, Finland; ^6^Department of Paediatric Cardiology, Evelina London Children's Hospital, Guy's & St. Thomas' NHS Trust, London, United Kingdom; ^7^School of Biomedical Engineering and Imaging Sciences, King's College London, London, United Kingdom; ^8^Minerva Foundation Institute for Medical Research, Biomedicum Helsinki, Helsinki, Finland; ^9^Department of Pediatrics, New Children's Hospital, University of Helsinki, Helsinki University Hospital, Helsinki, Finland

**Keywords:** cardiotoxicity, childhood cancer, longitudinal strain, speckle tracking, cardiovascular risk (CV risk)

## Abstract

**Background:** The majority of childhood cancer survivors (CCSs) have been exposed to cardiotoxic treatments and often present with modifiable cardiovascular risk factors. Our aim was to evaluate the value of left ventricular (LV) longitudinal strain for increasing the sensitivity of cardiac dysfunction detection among CCSs.

**Methods:** We combined two national cohorts: neuroblastoma and other childhood cancer survivors treated with anthracyclines. The final data consisted of 90 long-term CCSs exposed to anthracyclines and/or high-dose chemotherapy with autologous stem cell rescue and followed up for > 5 years and their controls (*n* = 86). LV longitudinal strain was assessed with speckle tracking (Qlab) and LV ejection fraction (EF) by three-dimensional echocardiography (3DE).

**Results:** Of the CCSs, 11% (10/90) had abnormal LV longitudinal strain (i.e., < -17.5%); of those, 70% (7/10) had normal 3DE LV EF. Multivariable linear model analysis demonstrated that follow-up time (*p* = 0.027), sex (*p* = 0.020), and BMI (*p* = 0.002) were significantly associated with LV longitudinal strain. Conversely, cardiac risk group, hypertension, age, cumulative anthracycline dose or exposure to chest radiation were not.

**Conclusion:** LV longitudinal strain is a more sensitive method than LV EF for the detection of cardiac dysfunction among CCSs. Therefore, LV longitudinal strain should be added to the screening panel, especially for those with modifiable cardiovascular risk factors.

## Introduction

The number of childhood cancer survivors (CCSs) reaching adulthood is increasing rapidly (1). In contrast, the doses of anthracyclines and chest irradiation have decreased in modern treatment protocols due their dose-dependent cardiotoxicity (2, 3). Some novel drugs, mainly used in adult oncology, also have cardiovascular side effects (4). Although cardiovascular damage coincides with exposure to the toxic treatment, clinical heart failure may not manifest until decades later. Modifiable cardiovascular risk factors, e.g., obesity, hypertension and dyslipidemia (5), are also common among CCSs and potentiate therapy-related adverse events (6, 7). As a result, a frequency of clinical heart failure ranging between 0 and 16% among CCSs has been reported post-treatment (8), and subclinical toxicity is even more prevalent (9). Thus, meticulous, long-term follow-up is of key importance for identifying patients at risk and for offering adequate treatment (3, 10). Consequently, a consensus recommendation on cardiomyopathy surveillance of CCSs has recently been put forth (1). However, the most commonly used echocardiographic methods, including ejection fraction (EF) and fractional shortening for the evaluation of systolic function, have some limitations with regard to sensitivity and accuracy (9, 11).

Speckle tracking-based LV longitudinal strain is a sensitive method for detecting decreased systolic function, even when the LV EF is still within normal limits (12). The subendocardial longitudinal fibers are prone to subtle injury because of their location; however, the LV EF comprises of radial, circumferential and longitudinal functions and thus deteriorates later than does longitudinal strain (13, 14). Left atrial (LA) strain is a promising tool for the detection of LV diastolic dysfunction (15). Guidelines recommend the use of global longitudinal LV strain together with LV EF for screening among adults during and after cancer treatment (4, 16). However, the inclusion of global longitudinal LV strain measurements has not yet been applied to children or adolescent protocols. Indeed, the best echocardiographic methods for the detection of asymptomatic cardiotoxicity remain to be determined.

The purpose of this study was to evaluate the additive value of strain imaging with regard to the sensitivity of cardiac dysfunction detection after childhood cancer.

## Materials and Methods

### Participants

**Figure 1:**
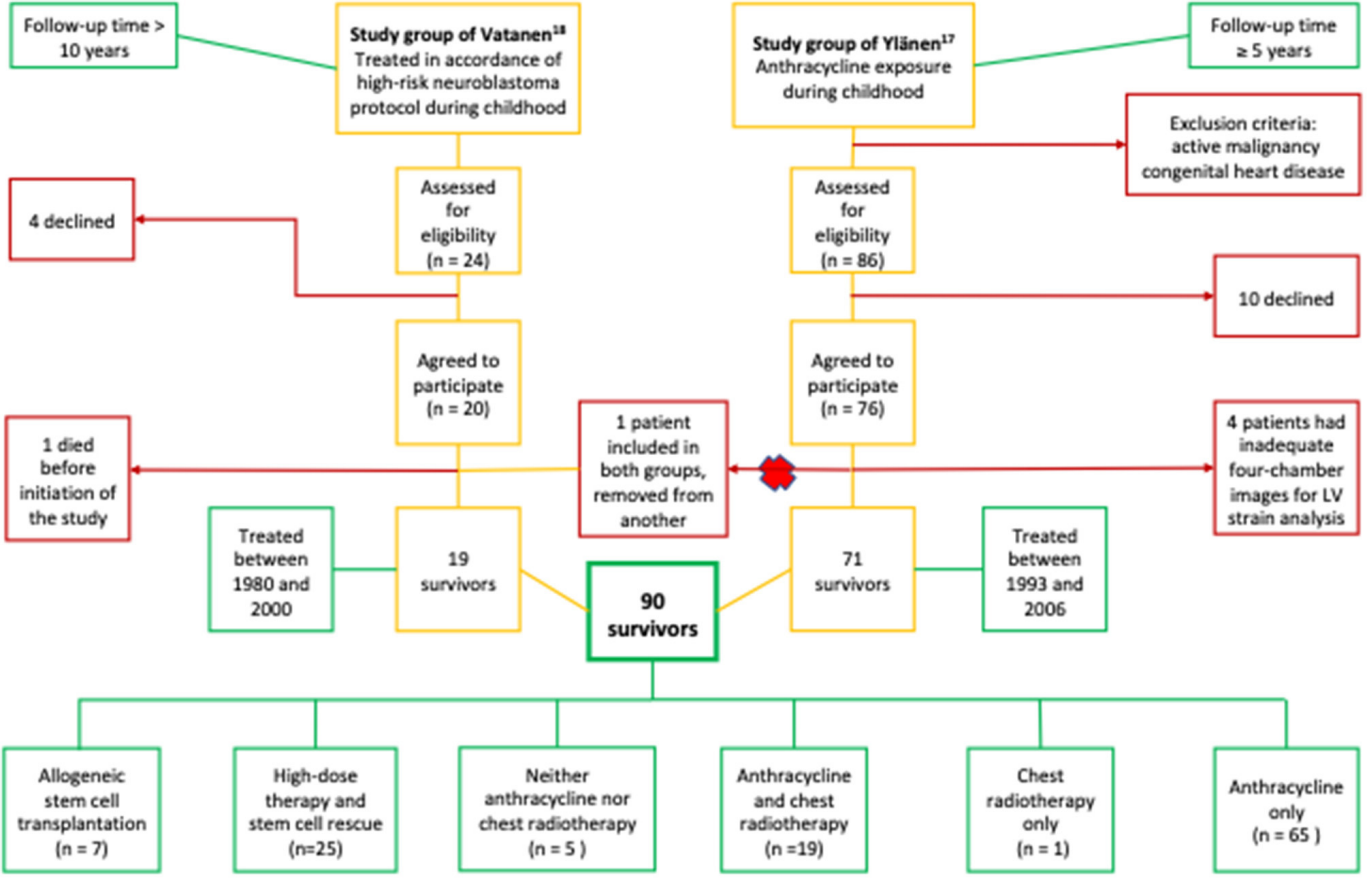
Study recruitment and combination flow chart. The number of study patients and their cancer therapy. The majority of the patients, 94% (85/90), were treated with anthracyclines and/or chest radiotherapy. The rest (5/90, 6%) were not exposed but had received high-dose therapy and stem cell rescue. Altogether, 28% (25/90) of the patients had received high-dose therapy and stem cell rescue, and 80% (20/25) of them were also exposed to anthracyclines and/or chest radiotherapy.

For this study, we combined data from two previously published cohorts of CCSs, firstly childhood cancer patients treated with anthracyclines, and secondly, a national cohort of neuroblastoma patients (Figure 1) (17, 18). The study group consisted of 95 long-term CCSs treated between 1980 and 2006 at the five university hospitals in Finland. The study patients were exposed to cardiotoxic treatments, e.g., anthracyclines and/or high-dose therapy with autologous stem cell rescue, and followed up for >5 years. None of the patients were given dexrazoxane during the survey. Six patients (6%) had heart failure treatment at the time of evaluation: 4 were treated with enalapril and two with enalapril combined with a beta-blocker. Stratification for long-term cardiac risk (i.e., cardiac risk groups) was performed according to the International Late Effects of Childhood Cancer Guideline Harmonization Group based on cumulative anthracycline and chest radiation doses (1). Healthy, age- and sex-matched controls were included for both study groups. The details of the clinical characteristics are shown in Table 1.

**Table 1:** Baseline characteristics of the study subjects.

**Variable**	**Survivors**	**Healthy controls**	***P***
*N*	90	86	
Age, years	16.0 ± 5.0	15.9 ± 4.9	0.898
Sex, *n* (%)			0.763
Female	49 (54)	49 (57)	
Male	41 (46)	37 (43)	
Height, (cm)	159 ± 15	164 ± 16	0.042^*^
Weight, (kg)	54 ± 17	57 ± 17	0.329
Body surface area (m^2^)	1.54 ± 0.31	1.60 ± 0.31	0.163
Body mass index^a^ (kg/m^2^)	22.5 ± 4.1	22.3 ± 3.2	0.735
Systolic blood pressure, mmHg (all age groups)	119 ± 14	111 ± 11	<0.001^*^
Diastolic blood pressure, mmHg (all age groups)	66 ± 9	63 ± 7	0.030^*^
**Malignancy diagnosis**, ***n*** **(%)**			
ALL	31 (34)		
Neuroblastoma	26 (29)		
AML	9 (10)		
Hodgkin disease	8 (9)		
Non-Hodgkin lymphoma	7 (8)		
Rhabdomyosarcoma	2 (2)		
Ewing	1 (1)		
Osteosarcoma	1 (1)		
Retinoblastoma	1 (1)		
Wilms' tumor	1 (1)		
Other	3 (3)		
Follow-up time post-treatment, years	8.1 (6.0–13.3)		
Anthracycline exposure, *n* (%)	84 (93)		
Cumulative anthracycline dose^b^ mg/m^2^	185 (120–292)		
Chest radiotherapy, *n* (%)	20 (22)		
TBI only	15		
Local radiotherapy	3		
TBI + local radiotherapy	2		
Cumulative chest radiotherapy doses (among 20 survivors exposed to chest radiotherapy), Gy	11 (10.0–18.3)		
Allogeneic SCT, *n* (%)	7 (8)		
Autologous SCT, *n* (%)	25 (28)		

* *Statistically significant*.

a *Body mass index (BMI) in adults, BMI according to age in children*.

b *Doxorubicin isotoxic equivalents*.

*SCT, stem cell transplantation; TBI, total body irradiation*.

The Research Ethics Committees of Helsinki and Tampere University Hospitals approved the study carried out in accordance with the Declaration of Helsinki. All the study subjects and their legal guardian(s) provided a written, informed consent.

### Blood Pressure

Right arm blood pressure was measured at rest according to guidelines. Stage one hypertension was defined as blood pressure ≥95th percentile or ≥130/80 mmHg (whichever was lower) for children under 13 years and ≥130/80 mmHg for those ≥13 years (19).

### Echocardiography

Echocardiographic examinations were performed by iE33 ultrasound (Philips, Andover, MA, USA) (17, 18) according to the American Society of Echocardiography (20, 21). The acquisition of the echocardiographic images was performed as described previously (17, 18).

Age-dependent reference values of the LV transmitral to septal mitral annular early diastolic velocity ratio (E/E′) and peak septal myocardial systolic velocity (S′) were used for subjects up to 18 years of age (22). LV E/E′ >10.0 and S′ <6.1 were considered abnormal for those over 19 years of age (23).

### Three-Dimensional Echocardiography

Analyses of three-dimensional echocardiographs were performed using commercial software (Qlab v9, Philips Medical Systems, Andover, MA, USA), as previously described (24). Briefly, the three-dimensional echocardiography (3DE) LV EF was calculated, and end-diastolic, end-systolic and stroke volumes were indexed to the body surface area (BSA). The LV end-systolic mass (LVMS) was normalized to height (meters) to a power of 2.7 (LVMSi), and the result expressed as g/m^2.7^.

### Qlab Peak Systolic Strain Analysis

LV longitudinal peak systolic strain from the four-chamber view was analyzed with Qlab (Philips Qlab, version 10.5, CMQ; Philips Healthcare, Bothell, WA, USA) and designated LV longitudinal strain. In the analysis, the points were placed at the edge of the mitral valve annulus on the septal and lateral sides and apex at end-diastole. The frame chosen by the program also automatically performed the rest of the tracing, which was manually checked and corrected if needed; the case was excluded if correction was not feasible. All systolic strain measurements were analyzed by one investigator blinded to all clinical and outcome data. Intraobserver analysis, performed using the Bland-Altman analysis (*n* = 10), showed mean of difference of 0.12 with limits of agreement between −1.08 and 1.32.

### Analysis of Diastolic Function and Atrial Parameters

Velocity vector imaging (VVI) of the left atrium (LA) was used to analyze LA indices and diastolic cardiac function. VVI analysis from the four-chamber view was performed with the VVI analysis program (Syngo USWP 3.0, Siemens Healthineers, Erlangen, Germany) as described (25). In short, manual tracing of the LA was performed using a single still frame in end-systole. Endocardial tracing began at the edge of the mitral valve annulus, extended to the base of the atrium and returned to the other edge of the annulus. The VVI algorithm calculated the velocity vectors for each frame of the cardiac cycle, displaying them for the complete loop. If the endocardial border was not traceable throughout the whole cardiac cycle, it was corrected manually. The parameters calculated were the LA area, LA fractional area change and VVI peak longitudinal strain for LA. The LA area was normalized to BSA, and the result expressed as cm^2^/m^2^.

### Statistical Methods

IBM SPSS Statistics version 24 (IBM Corp., Armonk, NY, USA) was used in this study. Categorical data are presented as frequencies and percentages, normally distributed continuous variables as the mean ± SD, and as median and interquartile ranges (IQR) in cases of non-normality. Categorical variables were compared with the chi-square or Fisher's exact-test. Means between two groups were compared using the independent samples *t*-test and medians with independent-samples using the Mann-Whitney *U*-test. Means were compared using one-way analysis of variance (ANOVA), and further pairwise comparisons between groups were performed with the Bonferroni method. When the homogeneity of variance was not met, Welch's ANOVA with Tamhane's-test was used for pairwise comparisons. Univariate associations of continuous variables associated with strain were analyzed with linear regression. A multivariable linear model was used to examine associations of multiple variables with strain. The cut-off value for the follow-up time to detect pathological strain was determined by a receiver operating characteristic (ROC) curve. The optimal cut-off value for follow-up time was chosen by using the Youden Index. A *p*-value < 0.05 was considered significant.

## Results

A total of 90 CCSs and 86 controls were included (Figure 1). Four survivors (4%) and 9 controls (9%) were excluded due to non-analyzable four-chamber views. The demographics of the subjects are listed in Table 1. The mean age of the CCSs at the time of the study was 16.0 ± 5.0 (range 7.2–30.1) years, and the median follow-up time post-treatment 8.1 (6.0–13.3) years. Of the survivors, 93% (84/90) were treated with anthracyclines, 28% (25/90) with high-dose therapy with autologous stem cell rescue, 8% (7/90) with allogeneic stem cell transplantation, and 22% (20/90) with radiation involving the heart [15 total body irradiation (TBI) only, 3 local radiotherapy and 2 both] (Figure 1). The normal strain value used for the Qlab was >-17.5% corresponding with the lower normal limits (mean – 1.96 SD) of our controls. The survivors had higher systolic and diastolic blood pressure than the controls, but BSA and body mass index (BMI) for children according to age (26) did not differ between the two groups (Table 1).

### LV Study Group Demographics

The baseline characteristics are presented in Table 2. Two groups were formed for the analyses: group S1 consisted of survivors with abnormal Qlab LV longitudinal strain (≤ -17.5%) (*n* = 10), and S2 of those with normal Qlab LV longitudinal strain (>-17.5%) (*n* = 80). Group C3 included all the controls (*n* = 86). The age and cardiac risk group did not differ between the groups. Group S1 contained more females (90 vs. 50%, *p* = 0.019) and had a longer follow-up (median 14.4 vs. 8.0 years, *p* = 0.007) than group S2. More survivors in S1 underwent chest radiotherapy (60 vs. 18%, *p* = 0.007). Hypertension was more common among the survivors (S1: 30%; S2: 29%) than the controls (C3: 7%, *p* = 0.05 and <0.001, respectively). Blood pressure among those of adult age was evaluated as a continuous parameter between the groups: adult survivors in S2 (*n* =17) had a higher systolic (128 ± 13 vs. 117 ± 10, *p* = 0.010) but not diastolic blood pressure (73 ± 9 vs. 67 ± 6, *p* = 0.088) than adult controls (*n* = 23). For the adult survivors in S1, the systolic (125 ± 15) and diastolic (75 ± 10) blood pressure was slightly higher than for controls, but the differences were not statistically significant (*p* = 0.444 and 0.132).

**Table 2:** Baseline characteristics and echocardiographic parameters of the study subjects according to group.

**Variable**	**S1:** **Survivors with abnormal Qlab** **LV longitudinal strain (≤-17.5%)**	**S2:** **Survivors with normal Qlab** **LV longitudinal strain (>-17.5%)**	**C3:** **Healthy controls**	***P***
**Baseline characteristics**				
*N*	10	80	86	
Age, years	18.1 ± 5.5	15.7 ± 4.9	15.9 ± 4.9	0.343
Sex, *n* (%)				S1-S2: 0.019^*^
Female	9 (90)	40 (50)	49 (57)	
Male	1 (10)	40 (50)	37 (43)	
Follow-up time, years	14.4 (10.0–20.1)	8.0 (6.0–12.1)		0.007^*^
Cardiac risk group^a^, *n* (%)				0.654
No	0 (0)	5 (6)		
Low	1 (10)	3 (4)		
Moderate	5 (50)	42 (53)		
High	4 (40)	30 (38)		
Cumulative anthracycline dose^b^, mg/m^2^	120 (118–210)	209 (123–295)		0.101
Chest radiotherapy, *n* (%)	6 (60)	14 (18)		0.007^*^
TBI only	4	11		
Local	0	3		
TBI + local	2	0		
Cumulative chest radiotherapy doses (among survivors exposed), Gy	12 (10–24.4)	10 (10–14.1)		0.328
Body surface area (m^2^)	1.51 ± 0.14	1.54 ± 0.32	1.60 ± 0.31	0.214
Body mass index^c^ (kg/m^2^)	23.5 ± 3.6	22.4 ± 4.1	22.3 ± 3.2	0.627
Stage 1 hypertension, *n* (%)	3 (30)	23 (29)	6 (7)	S1-C3: 0.050^*^ S2-C3: <0.001^*^
**LV systolic function**				
3DE LV EF, %	57.7 ± 6.9	60.5 ± 4.7	63.0 ± 4.9	<0.001^*^ S1-C3: 0.005^*^ S2-C3: 0.004^*^
3DE LV EDV index, ml/m^2^	49.7 ± 13.4	55.0 ± 8.7	56.6 ± 11.0	0.112
3DE LV ESV index, ml/m^2^	21.0 ± 6.0	21.7 ± 4.6	21.0 ± 5.0	0.577
3DE LV SV index, ml/m^2^	28.8 ± 8.6	33.2 ± 5.5	35.6 ± 7.3	0.019^*^ S2-C3: 0.049^*^
3DE LV systolic mass index, g/m^2.7^	38.0 ± 8.3	32.3 ± 7.4	30.9 ± 7.0	0.013^*^ S1-C3: 0.012^*^
Qlab LV longitudinal strain, %	16.5 ± 0.7	21.3 ± 2.4	22.4 ± 2.3	<0.001^*^ S1-C3: <0.001^*^ S2-C3: 0.008^*^ S1-S2: <0.001^*^
TDI S′, cm/s	7.1 ± 0.9	7.4 ± 1.1	8.2 ± 1.3	<0.001^*^ S1-C3: 0.019^*^ S2-C3: <0.001^*^
TDI S′ < −2 SD, *n* (%)	1 (10)	7 (9)	0 (0)	S2-C3: 0.005^*^
**LV diastolic function**				
LV E/E'	10.5 ± 3.2	8.1 ± 2.3	7.2 ± 1.4	0.001^*^ S1-C3: 0.029^*^ S2-C3: 0.008^*^
LV E/E′> +2 SD, *n* (%)	6 (60)	16 (20)	4 (5)	S1-C3: <0.001^*^ S2-C3: 0.003^*^ S1-S2: 0.012^*^
**Left atrium**				
LA area index, cm^2^/m^2^	9.7 ± 1.5	9.9 ± 1.9	10.4 ± 1.9	0.098
LA FAC, %	48.6 ± 7.7	55.9 ± 9.2	55.5 ± 8.4	0.046^*^ S1-S2: 0.041^*^
LA longitudinal strain, %	34.5 ± 12.5	45.5 ± 14.4	44.4 ± 13.7	0.066

* *Statistically significant*.

a *Cardiac risk group according to International Late Effects of Childhood Cancer guideline Harmonization Group. High risk: anthracycline dose ≥250 mg/m^2^, chest radiation dose ≥35 Gy or combined anthracycline dose ≥100 mg/m^2^ together with chest radiation dose ≥15 Gy. Moderate risk: anthracycline doses 100 to <250 mg/m^2^ or chest radiation dose ≥15 to <35 Gy. Low risk anthracycline doses below 100 mg/m^2^*.

b *Doxorubicin isotoxic equivalents*.

c *Body mass index (BMI) in adults, BMI according to age in children*.

*E/E′ = transmitral to septal mitral annular early diastolic velocity ratio; EDV, end diastolic volume; EF, ejection fraction; ESV, end systolic volume; FAC, fractional area change; LA, left atrium; LV, left ventricle; S′, peak myocardial sustained systolic velocity; SV, stroke volume; TDI, tissue Doppler imaging; 3DE, three-dimensional echocardiography*.

### Cardiac Analysis

Echocardiographic characteristics are shown in Table 2. Of the CCSs, 11% (10/90) had abnormal Qlab LV longitudinal strain. Seven of the 10 S1 survivors had normal LV EF (>55%) despite decreased LV longitudinal systolic function. Nine percent of all the CCS with normal LV EF had abnormal Qlab LV longitudinal strain. Moreover, LV EF was lower in S1 and S2 than in the controls (57.7 ± 6.9, 60.5 ± 4.7 vs. 63.0 ± 4.9%, *p* = 0.005 and 0.004, respectively). Although LV end-diastolic and -systolic volume indexes did not differ between the groups, the LV stroke volume index was lower in S2 than in controls (33.2 ± 5.5 vs. 35.6 ± 7.3 ml/m^2^, *p* = 0.049). The LVMSi was also higher in S1 (38.0 ± 8.3 vs. 30.9 ± 7.0 g/m^2.7^, *p* = 0.012).

There was more diastolic dysfunction (an abnormally high LV E/E′) in S1 (60%) than in S2 (20%, *p* = 0.012) and controls (5%, *p* < 0.001). The LA area index did not differ between the groups. The LA fractional area change was lower in S1 than in S2 (48.6 ± 7.7 vs. 55.9 ± 9.2%, *p* = 0.041). Despite a trend for lower LA longitudinal strain in S1 than in S2 and C3 (34.5 ± 12.5 vs. 45.5 ± 14.4 and 44.4 ± 13.7%), the difference was not statistically significant (*p* = 0.066).

### Variables Associated With LV Longitudinal Strain

Variables associated with LV longitudinal strain were also evaluated (Table 3). According to the univariate analysis, a long follow-up time (*p* = 0.029), female sex (*p* = 0.031), high BMI (*p* = 0.036) and chest radiotherapy (*p* = 0.004) were significantly associated with low strain. Furthermore, follow-up time (*p* = 0.027), female sex (*p* = 0.020) and BMI (*p* = 0.002) remained significant variables in the multivariable linear model, whereas cardiac risk group, high blood pressure, age, cumulative anthracycline dose or exposure to chest radiation did not (Table 3).

**Table 3:** Variables associated with strain in the patient group; univariate and multivariable analyses.

		**Univariate analysis^b^**		**Multivariable analysis^c^**	
**Variable**	***N***	**Mean ± SD or β (SE)**	***P***	**Adjusted mean (SE) or β (SE)**	***P***
Age (years)	90	−0.086 (0.058)	0.143	0.043 (0.094)	0.646
Sex (m/f)			0.031^*^		0.020^*^
Male	41	21.46 ± 2.59		21.89 (0.75)	
Female	49	20.21 ± 2.78		20.56 (0.72)	
BMI, kg/m^2^	90	−0.151 (0.071)	0.036^*^	−0.215 (0.068)	0.002^*^
Follow-up time (years)	90	−0.106 (0.048)	0.029^*^	−0.199 (0.088)	0.027^*^
Cardiac risk group			0.491		0.222
No risk	5	21.68 ± 2.48		23.83 (1.83)	
Low	4	19.70 ± 3.39		20.48 (1.41)	
Intermediate	47	21.08 ± 2.94		20.94 (0.51)	
High	34	20.37 ± 2.46		19.64 (0.64)	
Cumulative anthracycline dose,^a^ mg/m^2^	90	−0.001 (0.003)	0.727	0.002 (0.005)	0.619
Chest radiotherapy			0.004^*^		0.370
No	70	21.22 ± 2.50		21.57 (0.59)	
Yes	20	19.26 ± 3.11		20.87 (0.93)	
Hypertension			0.291		0.527
Normal	63	20.96 ± 2.91		21.43 (0.70)	
High	26	20.27 ± 2.33		21.03 (0.78)	

* *Indicates statistically significant*.

*β indicates regression coefficient for one-unit increase in continuous variables*.

a *Doxorubicin isotoxic equivalents*.

b *The means between two groups were compared with the independent samples t-test and between cardiac risk groups with the one-way ANOVA*.

*Linear regression was used to evaluate the association of continuous variable with strain*.

c *Multivariable linear model*.

There was a significant difference in the median follow-up time (14.4 years among the CCS with an abnormal strain vs. 8.0 years for those with a normal strain, *p* = 0.007). Females (9 of 49, 18.4%) had more abnormal strain (i.e., ≤ -17.5%) than males (1 of 41, 2.4%, *p* = 0.019). Patients exposed to chest radiotherapy (6 of 20, 30.0%) also had more abnormal strain than those not exposed (4 of 70, 5.7%, *p* = 0.007).

The area under the curve (AUC) for the follow-up time to detect pathological strain (i.e., ≤ -17.5%) was 0.76 (95% CI 0.63–0.89). The critical cut-off value for the follow-up time to increase the risk of abnormal strain was ≥ 9.24 years (sensitivity 0.90 and specificity 0.60).

## Discussion

Our study demonstrates that among CCSs with abnormal LV longitudinal strain, 70% had normal LV EF, suggesting that decreased LV longitudinal strain may emerge as a more sensitive marker of cardiotoxicity than LV EF following treatment for childhood cancer.

Our results are in line with recent data on adults showing abnormal LV longitudinal strain in 28% of CCSs despite normal LV EF (27). For adults, speckle tracking–based strain imaging has been well-validated for the measurement of LV deformation and recommended for echocardiographic, functional follow-up (1, 4, 16). However, the role of myocardial strain imaging among children is less well-established. In agreement with our results, several studies have found that the global systolic function and LV longitudinal strain are reduced among CCSs (2, 28–31), though these factors have yet to be proven to be more sensitive than LV EF in the early follow-up.

In this study, we report a decrease in strain in a subgroup of patients with preserved EF. This finding could not be explained by the patient age as a confounding variable, as it did not show a significant correlation with reduced longitudinal strain, either in univariate or multivariable analysis. In addition, although some level of decrease in the strain values has recently been shown to occur due to aging, in a recent study (32), any significant decrease in LV global longitudinal strain did not occur until the 8th decade of life and thus did not potentially affect the patients of our young study groups. This underlines the fact that the observed reduction in LV longitudinal strain among the CCSs is not based on increased age alone but instead on accumulated follow-up time after the original cancer treatment. Similarly, and importantly, male sex is known to be associated with lower longitudinal, circumferential, and radial strain (33), whereas in our study, the S1 patients with reduced strain with preserved EF were predominantly females. This further reduces the possibility of sex explaining the observed phenomenon of strain reduction in this population. Instead, females appear to be at higher risk of deleterious cardiotoxic effects of cytotoxic drugs.

Anthracycline toxicity and secondary cardiovascular risk factors mainly affect the subendocardial fibers (13, 14, 32), contributing negatively to longitudinal shortening prior to the reduction in LV EF. For example, patients with heart failure and preserved EF compensate for the reduction in longitudinal shortening by increasing twist to maintain normal EF (34). Thus, strain imaging has been proposed as a more sensitive technique to detect myocardial damage than LV EF. Early detection of cardiac failure development may be valuable for treatment with early interventions being considered more efficient than measures taken after abnormal EF is detected or clinical symptoms of dysfunction manifest (3, 16, 31, 35).

The value of LV longitudinal strain in detecting early myocardial dysfunction in cohort studies depends on how many events are assumed to occur simultaneously in the general population. The proportion of abnormal LV functional findings appears to depend on the length of follow-up (2, 6, 27, 31). In our cohort (median follow-up time 8.1; IQR 6.0–13.3 years), the proportion of survivors with preserved LV EF but with abnormal strain was 9%, lower than that reported by two adult studies [28% among those with normal LV EF after a mean follow-up time of 21.6 ± 7.9 years (27) or a median time from diagnosis of 23 years (range 10–48 years) (2)].

However, our results are comparable, albeit clearer, than those reported by others. For example, Slieker et al. (31) recently found reduced longitudinal strain in 7.7% of their 546 CCSs [median time since last anthracycline treatment, 7.9 (IQR, 5.6–10.6) years]. Similarly, three other studies reported significant decreases in the longitudinal function but did not report the prevalence of abnormal strain (median follow-up time, 5.2–13.2 years) (30, 34, 36).

The risk of cardiac dysfunction has been shown to increase with time (3), in line with our results. Similarly, the recent differing report (31) on the role of LV longitudinal strain in the follow-up of CCSs seems to be impacted by a shorter follow-up time than in our study demonstrating cardiac dysfunction more likely to be detectable by pathological strain when the follow-up time exceeds 9 years (sensitivity 0.90, specificity 0.60). Our results thus indicate that the longitudinal strain putatively offers a potent tool for risk assessment among the CCSs, especially early on and beyond the first decade of follow-up.

Our data are in line with those of Christiansen et al. (27) showing exposure to chest radiotherapy to be more common among the CCSs with abnormal LV longitudinal strain than others. However, in our study, the anthracycline dose did not correlate with the reduced longitudinal strain, again in line with the data of Slieker et al. (31), most likely indicating the absence of a safe dose of anthracyclines among the CCSs.

Both of our CCS groups (S1 with abnormal strain and S2 with normal strain) had a lower LV EF than controls (C3). A similar trend was also observed for the TDI S′, an additional sensitive marker of systolic function. The most common and best-established form of anthracycline cardiomyopathy indeed resembles dilated cardiomyopathy, with a thin-walled, large LV and low EF (3).

Our data further show and support those of others that the CCSs are at increased risk of modifiable cardiovascular risk factors such as hypertension and obesity (7). Hypertension induces LV hypertrophy and thus increases LV EF but possibly decreases stroke volume. Consequently, as an early marker of systolic dysfunction, the CCSs may have decreased LV longitudinal function with preserved EF. In our study, those with abnormal LV longitudinal strain (S1) had a higher LV systolic mass index and more hypertension than controls, illustrating this phenomenon. A large study on adult CCSs has shown that survivors with metabolic syndrome are twice as likely to have abnormal global longitudinal strain. Each individual component of the metabolic syndrome increases the risk, but without a higher risk for abnormal LV EF (2). Therefore, cancer treatment-related cardiomyopathy should no longer be solely defined as dilated but rather as mixed and further associated with an increased burden of treatment-related, modifiable risk factors, including hypertension-related LV hypertrophy with abnormal longitudinal strain, as early markers of LV dysfunction with preserved EF. Indeed, cardiomyopathy risk groups based on the anthracycline and chest radiation doses consist of high-risk survivors at an early stage of follow-up (1), but with further follow-up, the risks become less well-delineated due to the impact of modifiable cardiovascular risk factors. The inclusion of LV longitudinal strain in the screening armamentarium might thus improve detection for survivors in the low and moderate cardiomyopathy risk groups but with increased risk of LV dysfunction.

Diastolic LV dysfunction often precedes the systolic. In our study, LV E/E′ as a marker of diastolic dysfunction was higher among survivors than controls and peaked in group S1. However, there was no difference in the LA area index between the groups, most likely reflecting the fact that atrial restriction prevents enlargement despite diastolic dysfunction. In addition, we observed a trend toward lower LA longitudinal strain in our group S1 compared with the others, even though the difference was not statistically significant. A lower LA fractional area change in S1 compared with S2 demonstrated the same. These results are in line with the study of Morris et al. (15), showing LA strain to be more sensitive than the volume index for detecting LV diastolic dysfunction among adult patients at risk. Nevertheless, LA strain is mostly a research tool, with analysis of the thin atrial wall sometimes being technically challenging.

An earlier study comparing the different systems showed small but statistically significant intervendor variation in the assessment of LV longitudinal strain (37). Thus, ideally, an ultrasound device from the same vendor should be used during the follow-up whenever possible. We studied LV longitudinal strain using the more readily employable Qlab system. Importantly, the lower normal limit for Qlab LV longitudinal strain for our controls was in line with the −2 SD value (i.e., −17.5%) derived from recently published pediatric reference values (38).

### Limitations

The most important limitation of this study was its cross-sectional nature, rendering future prospective studies important to confirm an increase in the risk of pathological longitudinal strain during and after the first decade of follow-up. Because we used previously acquired echocardiographs from our earlier studies, LV longitudinal strain from the four-chamber views was employed, as opposed to global longitudinal strain. Nonetheless, optimal four-chamber views are easy to obtain, and in addition, four-chamber longitudinal strain has good intra- and interobserver correlation (38) and thus is usually sufficient for daily use in practice. Our division of patients into subgroups may also be considered somewhat arbitrary. Yet, the limits chosen (i.e., EF > 55% and strain > −17.5%) to define clinical normal values were adapted from those, already-published and generally accepted publications, and remained well in line with our own defined −1,96 SD lower limit of values from the age- and sex-matched control population (20, 33, 38). Important to note, none of the patients were given the cardioprotectant dexrazoxane during the survey, eliminating its possible effect on the measured strain rate among the CCS.

## Conclusions

Healthcare providers should pay special attention to the modifiable cardiovascular risk factors among the CCSs, as they play a pivotal role in developing heart failure long-term.

To date, the CCSs at risk for developing cardiac problems may not be identified early enough when using LV EF alone. Indeed, our results indicate that longitudinal strain putatively offers a potent tool for the long-term risk assessment among CCSs beyond the first decade of follow-up. Especially for those with modifiable cardiovascular risk factors and LV hypertrophy with normal EF, LV longitudinal strain would beneficially contribute to the final decision-making already in the pediatric population and among young adults.

## Data Availability Statement

The original contributions presented in the study are included in the article/supplementary material, further inquiries can be directed to the corresponding author.

## Ethics Statement

The studies involving human participants were reviewed and approved by Helsinki University Hospital, Helsinki, Finland; Tampere University Hospital, Tampere, Finland. Written informed consent to participate in this study was provided by the participants' legal guardian/next of kin.

## Author Contributions

JN, KY, and TO designed the study, collected and analyzed the data, and wrote the manuscript. KV and TP participated in designing the study, analyzing the data, and writing the manuscript. AS, KP, SM, TS, KJ, and AE participated in analyzing the data and writing the manuscript. All authors contributed to the article and approved the submitted version.

## Funding

This work was financially supported the Blood Disease Research Foundation, Helsinki; the competitive research funding of the Tampere University Hospital (9L114 and 9N084); the EVO funds of the Helsinki, Tampere and Turku University Hospitals; the Emil Aaltonen Foundation; the Finnish Association of Hematology; the Finnish Cancer Foundation; the Kirsti and Tor Johansson's Heart and Cancer Foundation; the Finnish Cultural Foundation; the Finnish Cultural Foundation Pirkanmaa Regional Fund; the Finnish Medical Foundation; the Foundation for Pediatric Research; the National Graduate School of Clinical Investigation, Helsinki; the Scientific Foundation of the City of Tampere and the Väre Foundation for Pediatric Cancer.

## Conflict of Interest

The authors declare that the research was conducted in the absence of any commercial or financial relationships that could be construed as a potential conflict of interest.

## Publisher's Note

All claims expressed in this article are solely those of the authors and do not necessarily represent those of their affiliated organizations, or those of the publisher, the editors and the reviewers. Any product that may be evaluated in this article, or claim that may be made by its manufacturer, is not guaranteed or endorsed by the publisher.

## Abbreviations

BSA: body surface area
CCS: childhood cancer survivors
E/E′: transmitral to septal mitral annular early diastolic velocity ratio
EF: ejection fraction
LA: left atrial/atrium
LV: left ventricular
LVMSi: indexed left ventricular mass at end-systole
S′: peak myocardial systolic velocity
VVI: velocity vector imaging
3DE: three-dimensional echocardiography.

## References

[1] Armenian SH Hudson MM Mulder RL Chen MH Constine LS Dwyer M. Recommendations for cardiomyopathy surveillance for survivors of childhood cancer: a report from the International Late Effects of Childhood Cancer Guideline Harmonization Group. Lancet Oncol. (2015) 16:e123–36. 10.1016/S1470-2045(14)70409-7 25752563 PMC4485458

[2] Armstrong GT Joshi VM Ness KK Marwick TH Zhang N Srivastava D. Comprehensive echocardiographic detection of treatment-related cardiac dysfunction in adult survivors of childhood cancer: results from the St. Jude lifetime cohort study J Am Coll Cardiol. (2015) 65:2511–22. 10.1016/j.jacc.2015.04.013 26065990 PMC4539123

[3] Lipshultz SE Adams MJ Colan SD Constine LS Herman EH Hsu DT. Long-term cardiovascular toxicity in children, adolescents, and young adults who receive cancer therapy: pathophysiology, course, monitoring, management, prevention, and research directions. Circulation. (2013) 128:1927–95. 10.1161/CIR.0b013e3182a88099 24081971

[4] Zamorano JL Lancellotti P Rodriguez Muñoz D Aboyans V Asteggiano R Galderisi M. 2016 ESC Position Paper on cancer treatments and cardiovascular toxicity developed under the auspices of the ESC Committee for Practice Guidelines. Eur Heart J. (2016) 37:2768–801. 10.1093/eurheartj/ehw211 27567406

[5] Turer CB Brady TM de Ferranti SD. Obesity, hypertension, and dyslipidemia in childhood are key modifiable antecedents of adult cardiovascular disease: a call to action. Circulation. (2018) 137:1256–9. 10.1161/CIRCULATIONAHA.118.032531 29555708 PMC5863574

[6] Armenian SH Armstrong GT Aune G Chow EJ Ehrhardt MJ Ky B. Cardiovascular disease in survivors of childhood cancer: insights into epidemiology, pathophysiology, and prevention. J Clin Oncol. (2018) 36:2135–44. 10.1200/JCO.2017.76.3920 29874141 PMC6804893

[7] Sundholm JKM Suominen A Sarkola T Jahnukainen K. Early arterial intimal thickening and plaque is related with treatment regime and cardiovascular disease risk factors in young adults following childhood hematopoietic stem cell transplantation. J Clin Med. (2020) 9:2208. 10.3390/jcm9072208 32668566 PMC7408962

[8] Kremer LCM van Dalen EC Offringa M Voûte PA. Frequency and risk factors of anthracycline-induced clinical heart failure in children: a systematic review. Ann Oncol. (2002) 13:503–12. 10.1093/annonc/mdf118 12056699

[9] Kremer LCM van der Pal HJH Offringa M van Dalen EC Voûte PA. Frequency and risk factors of subclinical cardiotoxicity after anthracycline therapy in children: a systematic review. Ann Oncol. (2002) 13:819–29. 10.1093/annonc/mdf167 12123328

[10] Rossano JW Shaddy RE. Heart failure in children: etiology and treatment. J Pediatr. (2014) 165:228–33. 10.1016/j.jpeds.2014.04.055 24928699

[11] Armstrong GT Plana JC Zhang N Srivastava D Green DM Ness KK. Screening adult survivors of childhood cancer for cardiomyopathy: comparison of echocardiography and cardiac magnetic resonance imaging. J Clin Oncol. (2012) 30:2876–84. 10.1200/JCO.2011.40.3584 22802310 PMC3671529

[12] Chinali M Esposito C Grutter G Iacobelli R Toscano A D'Asaro MG. Cardiac dysfunction in children and young adults with heart transplantation: a comprehensive echocardiography study. J Heart Lung Transplant. (2017) 36:559–66. 10.1016/j.healun.2016.11.007 28041955

[13] Kang Y Xiao F Chen H Wang W Shen L Zhao H. Subclinical anthracycline-induced cardiotoxicity in the long - term follow-up of lymphoma survivors: a multi-layer speckle tracking analysis. Arq Bras Cardiol. (2018) 110:219–28. 10.5935/abc.20180042 29694546 PMC5898770

[14] Cameli M Mondillo S Solari M Righini FM Andrei V Contaldi C. Echocardiographic assessment of left ventricular systolic function: from ejection fraction to torsion. Heart Fail Rev. (2016) 21:77–94. 10.1007/s10741-015-9521-8 26712329

[15] Morris DA Belyavskiy E Aravind-Kumar R Kropf M Frydas A Braunauer K. Potential usefulness and clinical relevance of adding left atrial strain to left atrial volume index in the detection of left ventricular diastolic dysfunction. JACC Cardiovasc Imaging. (2018) 11:1405–15. 10.1016/j.jcmg.2017.07.029 29153567

[16] Plana JC Galderisi M Barac A Ewer MS Ky B Scherrer-Crosbie M. Expert consensus for multimodality imaging evaluation of adult patients during and after cancer therapy: a report from the American Society of Echocardiography and the European Association of Cardiovascular Imaging. J Am Soc Echocardiogr. (2014) 27:911–39. 10.1016/j.echo.2014.07.012 25172399

[17] Ylänen K Eerola A Vettenranta K Poutanen T. Speckle tracking echocardiography detects decreased cardiac longitudinal function in anthracycline-exposed survivors of childhood cancer. Eur J Pediatr. (2016) 175:1379–86. 10.1007/s00431-016-2776-9 27620626

[18] Vatanen A Ojala TH Sarkola T Turanlahti M Jahnukainen T Saarinen-Pihkala UM. Left ventricular mass and ambulatory blood pressure are increased in long-term survivors of childhood cancer after autologous SCT. Bone Marrow Transplant. (2016) 51:853–5. 10.1038/bmt.2015.355 26828907

[19] Flynn JT Kaelber DC Baker-Smith CM Blowey D Carroll AE Daniels SR. Clinical practice guideline for screening and management of high blood pressure in children and adolescents. Pediatrics. (2017) 140:e20171904. 10.1542/peds.2017-1904 28827377

[20] Lang RM Bierig M Devereux RB Flachskampf FA Foster E Pellikka PA. Recommendations for chamber quantification: a report from the American Society of Echocardiography's Guidelines and Standards Committee and the Chamber Quantification Writing Group, Developed in Conjunction with the European Association of Echocardiography, a Branch of the European Society of Cardiology. J Am Soc Echocardiogr. (2005) 18:1440–63. 10.1016/j.echo.2005.10.005 16376782

[21] Lopez L Colan SD Frommelt PC Ensing GJ Kendall K Younoszai AK. Recommendations for quantification methods during the performance of a pediatric echocardiogram: a report from the pediatric measurements writing group of the American Society of Echocardiography Pediatric and Congenital Heart Disease Council. J Am Soc Echocardiogr. (2010) 23:465–95. 10.1016/j.echo.2010.03.019 20451803

[22] Eidem BW McMahon CJ Cohen RR Wu J Finkelshteyn I Kovalchin JP. Impact of cardiac growth on doppler tissue imaging velocities: a study in healthy children. J Am Soc Echocardiogr. (2003) 17:212–21. 10.1016/j.echo.2003.12.005 14981417

[23] Caballero L Kou S Dulgheru R Gonjilashvili N Athanassopoulos GD Barone D. Echocardiographic reference ranges for normal cardiac Doppler data: results from the NORRE Study. Eur Heart J Cardiovasc Imaging. (2015) 16:1031–41. 10.1093/ehjci/jev083 25896355

[24] Ojala T Mathur S Vatanen A Sinha MD Jahnukainen K Simpson J. Repeatability and agreement of real time three-dimensional echocardiography measurements of left ventricular mass and synchrony in young patients. Echocardiography. (2015) 32:522–7. 10.1111/echo.12672 24974764

[25] Ruotsalainen H Bellsham-Revell H Bell A Pihkala J Ojala T Simpson J. Right ventricular systolic function in hypoplastic left heart syndrome: a comparison of velocity vector imaging and magnetic resonance imaging. Eur Heart J Cardiovasc Imaging. (2016) 17:687–92. 10.1093/ehjci/jev196 26323279 PMC5841879

[26] Saari A Sankilampi U Hannila M-L Kiviniemi V Kesseli K Dunkel L. New Finnish growth references for children and adolescents aged 0 to 20 years: length/height-for-age, weight-for-length/height, and body mass index-for-age. Ann Med. (2011) 43:235–48. 10.3109/07853890.2010.515603 20854213

[27] Christiansen JR Massey R Dalen H Kanellopoulos A Hamre H Fosså SD. Utility of global longitudinal strain by echocardiography to detect left ventricular dysfunction in long-term adult survivors of childhood lymphoma and acute lymphoblastic leukemia. Am J Cardiol. (2016) 118:446–52. 10.1016/j.amjcard.2016.05.021 27296561

[28] Poterucha JT Kutty S Lindquist RK Li L Eidem BW. Changes in left ventricular longitudinal strain with anthracycline chemotherapy in adolescents precede subsequent decreased left ventricular ejection fraction. J Am Soc Echocardiogr. (2012) 25:733–40. 10.1016/j.echo.2012.04.007 22578518

[29] Kalam K Otahal P Marwick TH. Prognostic implications of global LV dysfunction: a systematic review and meta-analysis of global longitudinal strain and ejection fraction. Heart. (2014) 100:1673–80. 10.1136/heartjnl-2014-305538 24860005

[30] Cheung YF Hong WJ Chan GCF Wong SJ Ha SY. Left ventricular myocardial deformation and mechanical dyssynchrony in children with normal ventricular shortening fraction after anthracycline therapy. Heart. (2010) 96:1137–41. 10.1136/hrt.2010.194118 20511624

[31] Slieker MG Fackoury C Slorach C Hui W Friedberg MK Fan C-PS. Echocardiographic assessment of cardiac function in pediatric survivors of anthracycline-treated childhood cancer. Circ Cardiovasc Imaging. (2019) 12:e008869. 10.1161/CIRCIMAGING.119.008869 31826678

[32] Yoshida Y Nakanishi K Daimon M Ishiwata J Sawada N Hirokawa M. Alteration of cardiac performance and serum b-type natriuretic peptide level in healthy aging. J Am Coll Cardiol. (2019) 74:1789–800. 10.1016/j.jacc.2019.07.080 31582139

[33] Sugimoto T Dulgheru R Bernard A Ilardi F Contu L Addetia K. Echocardiographic reference ranges for normal left ventricular 2D strain: results from the EACVI NORRE study. Eur Heart J Cardiovasc Imaging. (2017) 18:833–40. 10.1093/ehjci/jex140 28637227

[34] Wang J Khoury DS Yue Y Torre-Amione G Nagueh SF. Preserved left ventricular twist and circumferential deformation, but depressed longitudinal and radial deformation in patients with diastolic heart failure. Eur Heart J. (2008) 29:1283–9. 10.1093/eurheartj/ehn141 18385117

[35] Obert P Gueugnon C Nottin S Vinet A Gayrard S Rupp T. Two-dimensional strain and twist by vector velocity imaging in adolescents with severe obesity. Obesity. (2012) 20:2397–405. 10.1038/oby.2012.111 22653310

[36] Thavendiranathan P Poulin F Lim KD Plana JC Woo A Marwick TH. Use of myocardial strain imaging by echocardiography for the early detection of cardiotoxicity in patients during and after cancer chemotherapy: a systematic review. J Am Coll Cardiol. (2014) 63:2751–68. 10.1016/j.jacc.2014.01.073 24703918

[37] Farsalinos KE Daraban AM Ünlü S Thomas JD Badano LP Voigt J-U. Head-to-head comparison of global longitudinal strain measurements among nine different vendors: the EACVI/ASE Inter-Vendor Comparison Study. J Am Soc Echocardiogr. (2015) 28:1171–81.e2. 10.1016/j.echo.2015.06.011 26209911

[38] Dallaire F Slorach C Bradley T Hui W Sarkola T Friedberg MK. Pediatric reference values and z score equations for left ventricular systolic strain measured by two-dimensional speckle-tracking echocardiography. J Am Soc Echocardiogr. (2016) 29:786–93.e8. 10.1016/j.echo.2016.03.018 27185223

